# Atlanta Academy of Medicine

**Published:** 1871-11

**Authors:** J. T. Johnson

**Affiliations:** Reporter


### ATLANTA ACADEMY OF MEDICINE.

J. T. JOHNSON, M.D., REPORTER.

OCTOBER 21st, 1871.

The President, Dr. J. P. Logan, in the chair.

### REPORTS OF CASES.

Under this call, Dr. W. N. Judson details a case of obstruction of the bowels. It is of the more interest from the use of a remedy recently under discussion by the Academy, namely, effervescence by seidlitz powders injected into the rectum. In a previous discussion, some fears were expressed as to the danger of rupturing the bowel. This case throws some light upon it.

The patient was a negro man, æt. forty. First prescribed some compound cathartic pills, with no effect. Then, after constipation had continued for eight days, prescribed large doses of calomel, jalap and podophyllin; also enema of warm water and soap. Next day, prescribed two seidlitz powders by the mouth and three by the rectum—some little mucus following their use. On the next day, the abdomen was still hard and distended. Directed a cathartic per orem, and five seidlitz powders to be injected; also, warm poultices to the abdomen. The next day, used seven seidlitz powders. On the day following, two drops of croton oil were given. Again, the next day, used ten seidlitz powders, injecting them himself. There was no difficulty or trouble from it, though there seemed to be a considerable commotion in the intestines. The patient died in a day or two. Throughout the disease, there was great pain in the abdomen. The pulse was normal from the beginning to within twelve hours of death. An examination was made by Drs. D'Alvigny and J. T. Johnson. Dr. Johnson being present, will request him to state its results.

Dr. Johnson stated that the examination was, to him, an unsatisfactory one. The precise location of the difficulty was not ascertained, but it was near the junction of the transverse and descending colon. The intestine above that point, or its neighborhood, was distended with fæcal matter; below, it was empty. The first part of the examination was conducted by Dr. D'Alvigny. The intestine was torn by the traction incident to the manipulation. This interfered with determining the exact location of the trouble; still, we were satisfied there was no invagination or impaction. The man had received a stab with a knife, some months ago, which penetrated just below the diaphragm. The wound was entirely healed externally, but internally there were evidences of a recent abscess, and inflammation of the neighboring peritoneum. Just in this region was the obstruction of the bowel. The bowel, for some distance, was disorganized or softened, and hence its easy division. There seemed to be no inflammatory redness of the bowel itself. The belief was, that some constricting band, resulting from the peritonitis, must have caused the obstruction. Thinks the case goes to prove that the bowel is not easily ruptured by effervescence, else such must have been the case in this instance, as it could be easily torn with the finger.

Dr. W. F. Westmoreland.—The man evidently died from the obstruction. There was no general peritonitis to account for the result. The pain and prostration resulting from obstruction, and lasting long enough, will kill anybody. It remains to determine positively the cause of the constipation.

Dr. J. S. Wilson.—What could have been the immediate cause of death? It seems that there was no general peritonitis to account for it.

Dr. W. F. Westmoreland has recently seen some cases of an interesting nature. One was in his own practice, and two in that of Dr. J. M. Boring. The patients were little girls—his case aged two, and Dr. Boring's three and five—who were affected with leucorrhœa. His case was of a lymphatic temperament, but in good health. In one of Dr. Boring's cases, but in neither of the other two, there were ascarides, as shown by the use of milk and turpentine. There was a continuous discharge from the vagina, resulting in excoriation if cleanliness was not observed. What is the pathology?—what the cure?

Dr. Harden.—Was there pain on urination?

Dr. W. F. Westmoreland.—All the surrounding parts were inflamed, though there was no pain in urinating.

Dr. Cumming.—Leucorrhœa often results from worms. You state that there were ascarides in one of the cases. Was similar trial made in the other two, and none observed?

Dr. Westmoreland.—There were none present.

Dr. Harden.—It may have been gonorrhœa. The nurse might have infected the child with it, or with leucorrhœa. Any mucous membrane may take on inflammation. Witness balanitis in the male; this may result from contact with leucorrhœal discharge.

Dr. Westmoreland.—It is no gonorrhœa. Has been more than once arrested completely, but will return at the end of five or six days.

Dr. Wilson.—Vaginitis sometives prevails as an epidemic, as in hospitals. As to the cause of this leucorrhœa, children are subject to the same *general* causes as adults.

Dr. Cumming.—Any relationship between the patients?

Dr. Westmoreland.—Dr. Boring's two cases were sisters; no relationship with the other.

Dr. Cumming.—Did Dr. Boring's cases occur synchronously?

Dr. Westmoreland.—I don't know.

Dr. Cumming.—What has been the duration?

Dr. Westmoreland.—Five or six months. Would like to hear from the President on this subject.

Dr. Logan.—Has no views with which to enlighten him, though has had some unsatisfactory experience of this nature. Doesn't understand why we should not as reasonably expect an inflammation here as in other parts—as eyes, nostrils, etc.

Dr. Harden.—Can understand this as being similar in its nature to strumous ophthalmia.

Dr. Cumming.—While we have hundreds and thousands of cases of strumous ophthalmia, this is a rare disease. Has met with several similar cases, but never with one in which the pin worms were not found. If not from this cause, then he has no knowledge of the disease. The books do not mention it. We may sometimes have cases of inoculation, but they are rare. These children should be placed upon a restorative treatment.

